# Supplementary material for: A Highly Sensitive and Selective ppb-Level Acetone Sensor Based on a Pt-Doped 3D Porous SnO2 Hierarchical Structure
Source: Sensors (Basel). 2020 Feb 19;20(4):1150. doi: 10.3390/s20041150 (PMC7070610; doi:10.3390/s20041150)
Supplement: Supplementary file 1 [file sensors-20-01150-s001.pdf]

Support Information

A highly sensitive and selective ppb-level acetone sensor based on a Pt-doped 3D porous SnO_2 hierarchical structure

Wenjing Quan, Xuefeng Hu, Xinjie Min, Junwen Qiu, Renbin Tian, Peng Ji, Weiwei Qin, Haixin Wang, Ting Pan, Suishi Cheng, Xiaoqiang Chen, Wei Zhang, Xiaorong Wang , and Hua Zheng

Figure S1

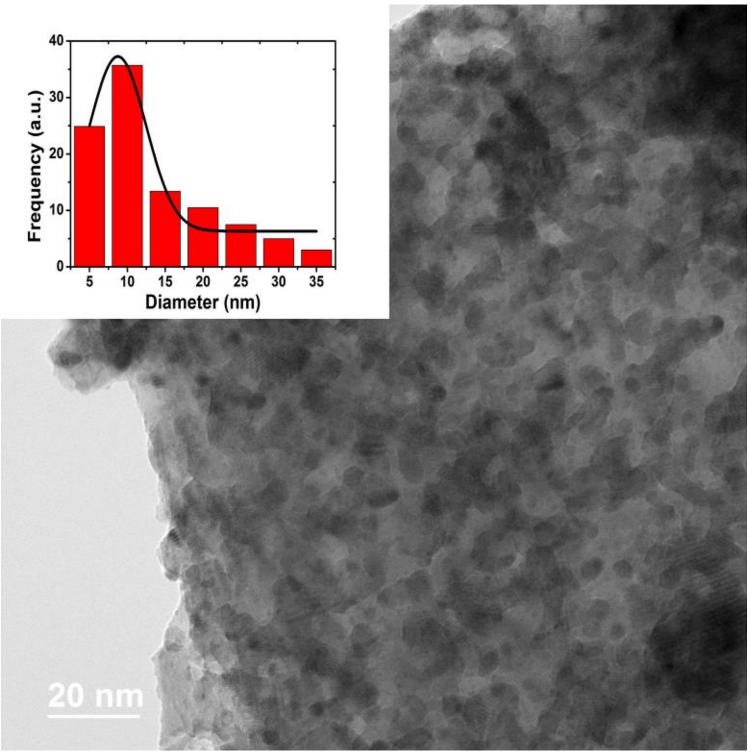

Figure S1. The size distribution of nanoparticles.

Figure S2

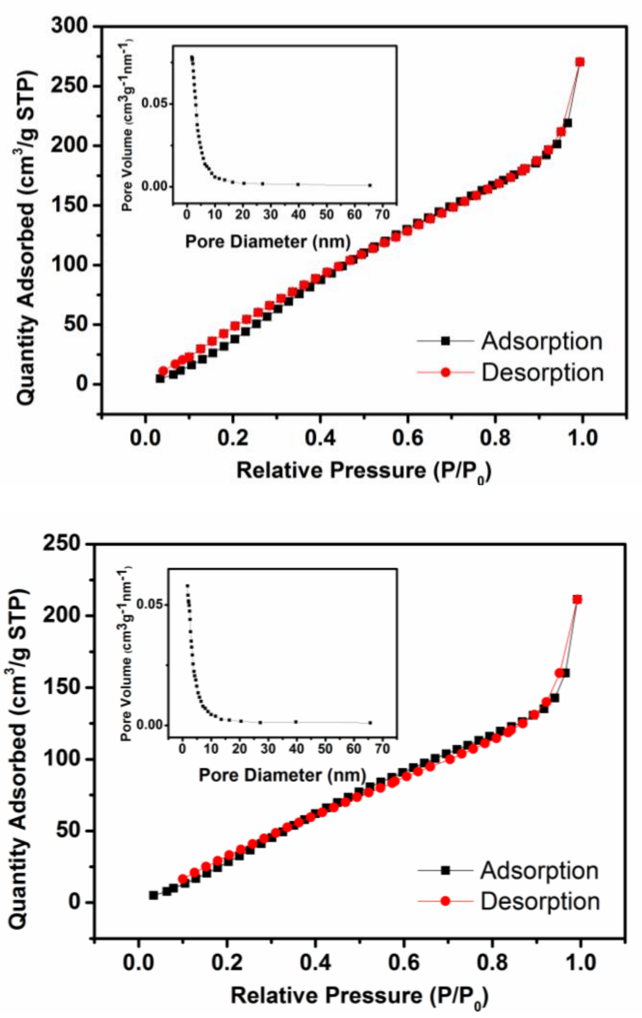

Figure S2. Nitrogen adsorption-desorption isotherms of pure SnO_2 and Pt-doped 3DS.

Growth of Pt-doped 3D Porous SnO_2

Based on the experimental observations and analysis, a possible growth mechanism of the hierarchical Pt-doped SnO_2 nanoflowers is proposed in Figure S3. A step-by-step reaction is listed in equations (1)-(4). The precursor $Sn(OH)_4^{2-}$ and $Pt(OH)_6^{2-}$ anions are formed from the Sn^{2+} ion of $SnCl_2 \cdot 2H_2O$, the $PtCl_6^{2-}$ ion of H_2PtCl_6 and the excess OH^- ions of NaOH, according to reactions (1) and (2). Salicylic acid can be used a chelating agent and easily bonds to form a stable complex compound with $Sn(OH)_4^{2-}$ in the solvent. The selective coordination of salicylic acid leads to the anisotropic growth of the SnO_2 crystals, which hinders the rapid formation of different orientations of nanosheets and thus facilitates the self-assembly of the nuclei into SnO_2 nanosheets to form SnO_2 nanoflowers. As the reaction proceeds, the reactions occur during the hydrothermal process according to reactions (3) and (4).

$$Sn^{2+} + 4\ OH^- \rightarrow Sn(OH)_4^{2-} \quad (1)$$

$$PtCl_6^{2-} + 6\ OH^- \rightarrow Pt(OH)_6^{2-} + 6\ Cl^- \quad (2)$$

$$Sn(OH)_4^{2-} \rightarrow SnO_2 + 2\ H_2O + 2\ e^- \quad (3)$$

$$Pt(OH)_6^{2-} \rightarrow PtO_x + H_2O + O^{2-} \quad (4)$$

Multiple pores form on the petals by postannealing the sample at 700°C. The thermal decomposition of the organic impurity (salicylic acid) and the release of gas into the confined space during the sintering process help to generate the porous structure.

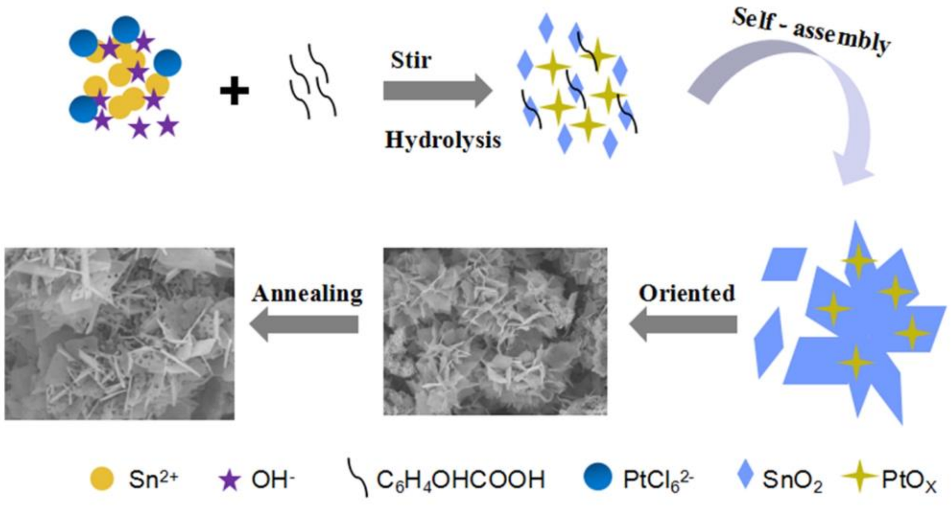

Figure S3. A possible evolution process of the SnO_2 nanoflowers.
